# Advanced Wearable Devices for Monitoring Sweat Biochemical Markers in Athletic Performance: A Comprehensive Review

**DOI:** 10.3390/bios14120574

**Published:** 2024-11-26

**Authors:** Graziana Assalve, Paola Lunetti, Alessandra Di Cagno, Ernesto William De Luca, Stefano Aldegheri, Vincenzo Zara, Alessandra Ferramosca

**Affiliations:** 1 Department of Experimental Medicine, University of Salento, 73100 Lecce, Italy; graziana.assalve@unisalento.it (G.A.); paola.lunetti@unisalento.it (P.L.); vincenzo.zara@unisalento.it (V.Z.); 2 Department of Human Sciences, Guglielmo Marconi University, 00193 Rome, Italy; alessandra.dicagno@uniroma4.it; 3 Department of Movement, Human and Health Sciences, University of Rome “Foro Italico”, 00135 Rome, Italy; 4 Department of Engineering Sciences, Guglielmo Marconi University, 00193 Rome, Italy; ew.deluca@unimarconi.it (E.W.D.L.); s.aldegheri@unimarconi.it (S.A.); 5 Institute of Technical and Business Information Systems, Otto-von-Guericke-University of Magdeburg, 39106 Magdeburg, Germany

**Keywords:** biosensors, cortisol, electrolytes, glucose, lactate, pH, sport

## Abstract

Wearable technology has advanced significantly, offering real-time monitoring of athletes’ physiological parameters and optimizing training and recovery strategies. Recent developments focus on biosensor devices capable of monitoring biochemical parameters in addition to physiological ones. These devices employ noninvasive methods such as sweat analysis, which reveals critical biomarkers like glucose, lactate, electrolytes, pH, and cortisol. These biomarkers provide valuable insights into an athlete’s energy use, hydration status, muscle function, and stress levels. Current technologies utilize both electrochemical and colorimetric methods for sweat analysis, with electrochemical methods providing higher precision despite potential signal interference. Wearable devices such as epidermal patches, temporary tattoos, and fabric-based sensors are preferred for their flexibility and unobtrusive nature compared to more rigid conventional wearables. Such devices leverage advanced materials and transmit real-time data to computers, tablets, or smartphones. These data would aid coaches and sports medical personnel in monitoring athletes’ health, optimizing diets, and developing training plans to enhance performance and reduce injuries.

## 1. Introduction

Wearable technology has transformed sports and health monitoring, offering real-time insights into physiological parameters for athletes and coaches. Unlike traditional, often invasive methods, wearable devices with biosensors provide a noninvasive, convenient solution for continuous monitoring during training and competition. These wearables are already used to track heart rate, blood pressure, and respiratory rate [1]. Monitoring physiological changes in real time is crucial for maintaining optimal athletic performance [2]. However, these devices do not allow coaches to quantify an athlete’s biochemical profile in real time [3]. Thus, there is a need to develop biosensors that measure biochemical markers like glucose, lactate, electrolytes, pH, and cortisol, which indicate physical exertion, fatigue, and mental well-being. These biochemical parameters are essential health indicators and are key to developing noninvasive wearable systems [4].

Human body fluids carry rich biological information, serving as valuable biomarkers for exercise monitoring. Blood has traditionally been the gold standard [5,6,7], but its invasive nature causes discomfort. Advances in technology have highlighted noninvasive fluids like sweat, tears, and saliva for their potential in exercise monitoring without affecting performance [8,9]. These fluids are ideal for real-time monitoring due to their noninvasive sampling methods. Saliva, in particular, is excellent due to its noninvasive nature and continuous supply [10]. Many biomarkers show a direct correlation between their concentrations in saliva and blood [11]. However, current devices have not yet achieved continuous biomarker measurements from saliva. The main drawback of using saliva for real-time measurements is the potential discomfort of integrating a sensor into a mouthguard, which is not standard in all sports [12].

Sweat is another ideal source for the continuous and noninvasive measurement of biomarkers [13]. Detecting biomarkers from eccrine sweat glands offers significant advantages, due to their abundance on the body, ease of access, and efficient sampling and detection [14]. During intense physical activity, eccrine sweat glands excrete fluids [14,15] containing electrolytes, metabolites, and hormones, which passively or actively enter sweat from nearby blood or interstitial fluids [16,17]. This allows for real-time molecular analysis of the body’s physiological state [18,19]. Additionally, analyte concentrations in sweat are much higher than in saliva and in most cases, they correlate with those in blood [20,21,22]. However, disadvantages include skin contamination and low sampling rates [13]. For this reason, wearables artificially induce sweating using a current, but care must be taken to avoid causing discomfort due to excessive stimulation, skin irritation, or localized heating, all of which can affect user compliance and the accuracy of biomarker readings.

Some of these devices have been tested on subjects with medical conditions, while others have been tested on healthy subjects during physical exertion, mostly indoors under controlled conditions.

For applicability in sports, biosensors should perform real-time monitoring, integrating biological sensing elements with transducers to detect and convert biological responses into electrical or colorimetric signals [23,24]. Monitoring specified biomarkers that provide real-time information on athletic condition is an effective support for optimizing training and enhancing athletes’ performance [3].

The selection of several biomarkers is determined by the needs related to the athlete’s characteristics (level, age, maturation, and goals) and the characteristics of the sports performance (volume, intensity, specificity) [25]. Examining electrolyte levels in sweat helps to prevent muscle cramps, dehydration, and performance issues during intense physical activity [3,26]. Lactate is a key indicator of muscle fatigue and the anaerobic threshold: tracking it allows for the adjustment of training intensity and the optimization of recovery [27]. Glucose regulation during exercise is influenced by the type, duration, and intensity of physical activity [28] and provides useful information about the athlete’s energy levels [28]. Elevated cortisol levels in sweat represent physical and mental stress, indicating the need to adjust training recovery and avoid overloads and injuries [29]. Hydrogen ions (H^+^), which affect sweat pH, can provide valuable information on the athlete’s metabolic state and acid–base balance [30]. Supervising these biomarkers could allow sports practitioners involved in competitive performance training programming to develop accurate work plans during different training periods: primarily by managing the load–recovery ratio effectively using an individualized method based on biochemical responses to the load [31]. This approach optimizes the administration of the load specifically tailored for each athlete and each specialty.

Concurrently, identifying training methods, selected using objective data, from monitoring the progressive improvement of various performance components will optimize interventions across different training phases of a sports season.

Clinical trials are crucial, especially for sweat-measuring biosensors, as climate and temperature significantly affect biomarker detection. For instance, athletes in humid environments produce more sweat than those in cold climates, and swimmers sweat less than other athletes [3,32].

This review aims to provide insights into the diverse functionalities and future directions of biosensor technology in sports monitoring, examining existing literature on wearable equipment for measuring sweat biochemical parameters applicable in sports.

## 2. Literature Search Methods

**Figure 1:**
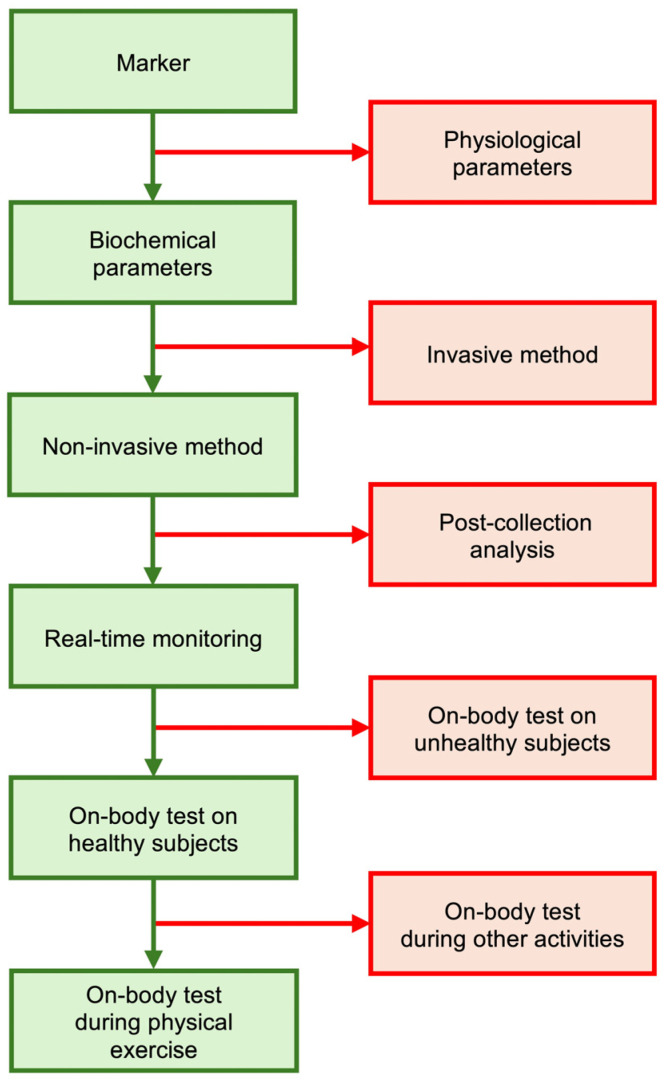
Literature search methods. Flowchart of the selection (in green) and exclusion criteria (in red) for literature review.

A thorough literature search was conducted across electronic databases, including PubMed, Scopus, and Web of Science, targeting articles published between 1999 and 2024. The search utilized keywords relevant to wearable technology, biochemical parameters, and sports (“wearable sensors”, “biochemical profile”, “athletic performance”). Studies published until April 2024 were screened based on criteria including the type of measured parameter, investigation method, analysis strategy, subject categories for in vivo tests, and activities during on-body tests (Figure 1).

We excluded articles related to physiological parameter monitoring and invasive or minimally invasive biochemical parameter monitoring in blood or interstitial fluid due to the potential disruption of athletic performance. Further review of titles and abstracts led to the exclusion of studies that did not report on biosensors capable of real-time monitoring or tested the technology on subjects with pathologies. Additional exclusions were made for studies lacking indoor tests or clinical trials on healthy subjects during physical activity. From the initial search, 57 studies met the inclusion criteria. Selected articles focused on wearable devices tested for assessing biochemical parameters in healthy subjects during physical exercise and were written in English; those addressing non-biochemical parameters or non-wearable and uncomfortable devices were excluded. Data extraction included biochemical parameters measured, the biological fluid in which the biomarker is measured, wearable device specifications (type of wearable platform and biosensor method), and the outcomes of indoor tests and clinical trials.

## 3. Glucose Monitoring for Fatigue Assessment

Glucose is the primary energy source in the human body, metabolized through glycolysis in cell cytoplasm. Monitoring glucose levels is crucial for managing fatigue in athletes, as adequate glucose transport to muscles during exercise is essential to prevent deficits that can impact performance [33,34]. Real-time glucose monitoring biosensors for sweat suitable for sports have been developed (Table 1).

Firstly, researchers have investigated the correlation between blood and sweat glucose levels [49]. Due to the highly vascularized nature of eccrine glands and the osmotic pressure-driven mechanism of sweat secretion, changes in blood glucose concentrations affect sweat glucose concentrations [50]. Sweat glucose ranges from 10 to 200 µM, which is 50 to 1000 times lower than blood glucose, depending on the sweat production rate. Despite this significant difference, the trends in glucose concentrations in both fluids are comparable. Additionally, there is a ten-minute delay between changes in blood glucose levels and corresponding changes in sweat glucose levels [30,51].

### 3.1. Electrochemical Devices

Amperometric and voltammetric techniques are commonly used for detecting biomarkers like glucose, as they involve enzyme-catalyzed redox reactions. These sensors typically comprise three main components: an enzyme that catalyzes the redox reaction of the target analyte, an electronic mediator that facilitates electron transfer between the enzyme and the electrode, and an electrochemical transducer that converts the biochemical reaction into an electrical signal. The most used enzymes are glucose oxidase (GOx) and glucose dehydrogenase (GDH). GOx, which uses the flavin adenine dinucleotide (FAD) cofactor, oxidizes glucose to yield gluconic acid and hydrogen peroxide; GDH, using cofactors like pyrroloquinoline quinone (PQQ) or nicotinamide adenine dinucleotide (NAD), also oxidizes glucose but is independent of the oxygen concentration. However, GDH can react with other sugars, whereas GOx is highly specific to glucose and more stable [52]. The intensity of the current recorded during the oxidation or reduction in the redox-active species is proportional to the analyte concentration, as described by Faraday’s law. Chronoamperometry, where a fixed potential is applied between the working and reference electrodes, allows for a current response governed by diffusion, which can be quantified using the Cottrell equation [53].

Epidermal patches are commonly used wearable platforms for sweat analysis because they leverage microfluidics or iontophoresis to address potential sample contamination and reduce sample volume issues. Microfluidic platforms collect sweat from the skin’s surface by connecting to eccrine sweat glands from various body parts, shortening sampling times and ensuring efficient glucose transport to the sensing electrodes. Waterproof microfluidic devices have also been developed to adhere to the skin and store and analyze sweat even underwater. These designs prevent contamination from aquatic environments while allowing sweat to flow to the sensor [54].

Over the past decade, significant progress has been made in developing wearable electrochemical sensors for noninvasive sweat glucose detection. Enzyme-based electrochemical glucose sensors are highly selective and sensitive, making them the most well-developed [35,36,37,38,39,40,42,43,44,45,52,55,56]. However, several challenges hinder their reliability, including enzyme deactivation due to temperature fluctuations, interference mitigation limitations, and the impact of environmental factors like temperature and humidity [57]. A notable advancement is the hybridization of a textile microfluidic component with a non-enzymatic nanoporous gold-based sensor, resulting in a fully stretchable sweat-sensing patch for glucose detection with high sensitivity and durability [41].

#### 3.1.1. Patch-Type Devices

**Figure 2:**
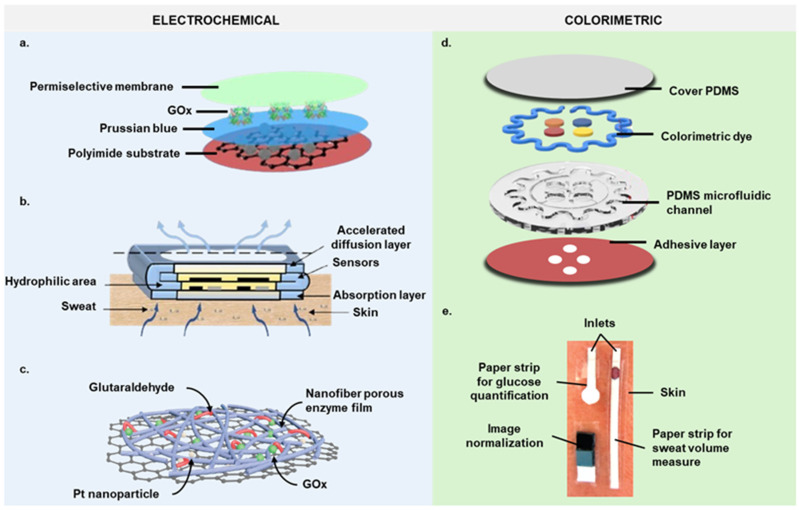
Structural anatomy of the most representative glucose biosensors. Schematic illustration of (**a**) a hybridized nanoporous carbon-reinforced 3D graphene-based epidermal patch [37], (**b**) the HIS paper [43], (**c**) the composition of the PEN membrane [45], (**d**) an epidermal microfluidic biosensor integrated with flexible electronics [24], and (**e**) a photograph showing a real application of a device made of paper strips [48].

New soft, skin-mounted microchip fluidic detection systems combine the advantages of electrochemical epidermal sensing and electrochemical microchip detectors, minimizing direct skin contact and sample evaporation. These advanced epidermal microfluidic devices, designed as patches, feature a flexible sensor array printed on carbon-based materials like polydimethylsiloxane (PDMS), polyimide (PI), three-dimensional porous graphene, Nafion, and styrene-ethylene-butylene-styrene block copolymer (SEBS) using screen printing technology (Figure 2a). The flexible microchip device adheres easily to the epidermis, ensuring conformal contact with the sweat pores to rapidly channel sweat towards the sensing reservoir while withstanding the repetitive mechanical deformations experienced by the wearer [35,36,37,38,39,40].

#### 3.1.2. Paper-Based Devices

All-paper sweat sensors for glucose offer advantages like affordability, compact size, and ease of use. These sensors can attach directly to the skin for comfort and convenience [58]. They enable direct electron transfer between glucose oxidase and the anodes, enhancing sensitivity and accuracy with a large anode surface area [59,60]. This improves signal strength and reliability, making them effective for real-time glucose monitoring in sweat [42,43].

Cho et al. integrated a paper-based glucose/oxygen enzymatic fuel cell into a Band-Aid for close skin contact. Though a separate detector was still needed, the setup was simple and inexpensive [42]. Similarly, a wearable platform for continuous glucose analysis in sweat was developed using highly integrated sensing paper (HIS paper). This paper incorporates two-dimensional MXene/methylene blue (Ti_3_C_2_T_x_/MB) as the active material and hydrophobic protecting wax and foldable all-paper substrates to develop a sweat analysis patch. The components are printed on paper and folded into a three-dimensional structure, enhancing sweat absorption and detection precision while preventing discomfort from sweat contact (Figure 2b) [43].

#### 3.1.3. Other Wearable Platforms

A wearable flexible integrated sensing array (FISA) for screening glucose and other biomarkers in sweat combines commercially available integrated circuit technologies with flexible sensors on plastic substrates. The sensors, fabricated on flexible polyethylene terephthalate (PET), ensure stable contact with the skin. The flexible printed circuit board (FPCB) technology includes signal conditioning, processing, and wireless transmission using common integrated circuit components. The entire system is flexible and can be worn as a smart wristband or forehead band. Studies show that biosensors maintain a consistent sensitivity for at least four weeks [44].

Enzyme stability is enhanced by immobilizing enzymes into a porous enzymatic network (PEN) membrane, attached to platinum nanoparticles (PtNPs)/graphene nanocomposite film (Figure 2c). The partially reduced graphene oxide provides abundant binding sites for the PEN membrane, offering a large surface area for biochemical reactions. This design ensures glucose sensor stability, which is then integrated into a soft forehead band for real-time sweat analysis during exercise. Signals are wirelessly transmitted to a Bluetooth-enabled mobile handset [45].

### 3.2. Colorimetric Devices

Colorimetric assays offer an alternative to electrochemical methods, eliminating the need for power supplies and external hardware [3]. Additionally, mobile colorimetric wearable biosensors for monitoring glucose in sweat use smartphone cameras for reading signals, ideal for self-monitoring [24,30,46,48]. Despite advancements, there is a need for simple, cost-effective colorimetric sweat detection chips suitable for mass production, especially in sports [30]. However, these biosensors typically focus on a limited range of biomarkers and provide semi-quantitative results rather than precise numerical values [46].

#### 3.2.1. Patch-Type Devices

In 2016, researchers developed a thin and soft closed microfluidic system for reliable sweat harvesting directly from skin pores. This device routes sweat through channels for multiparametric sensing of various markers [24]. It uses biocompatible adhesives, is flexible and stretchable, and can be mounted on multiple body locations without irritation. This prevents leakage and ensures sample integrity. The device detects total sweat glucose concentration colorimetrically, with wireless data transmission for immediate access to results (Figure 2d) [24]. Tested in variable conditions during an outdoor cycling race, it showed real-world performance without adhesion loss or fluid leakage and no discomfort at the skin interface [24].

Superwettable band-based biosensors represent another advance in noninvasive biofluid analysis. They use superhydrophobic–superhydrophilic microarrays and nanodendritic colorimetric biosensors for precise sweat collection and reliable analysis. These bands effectively sensed raw sweat on the wrist of a volunteer and evaluated it in real time via portable cellphone screening. However, glucose levels in collected sweat were low, providing only semi-quantitative data [46].

To enhance colorimetric devices, Bandodkar et al. developed a battery-free device integrating electronic, microfluidic, and colorimetric assays. Lighter, cheaper, and smaller than existing options, it securely adheres to the skin and offers continuous monitoring of sweat loss and chemistry during physical activity. This device combines electronic and microfluidic technologies for versatile, effective noninvasive sweat analysis [47].

#### 3.2.2. Paper-Based Devices

Vaquer et al. proposed a paper-based biosensor as an innovative alternative to polymer colorimetric devices. This biosensor includes a sweat volume sensor and a color chart for signal correction, offering environmental friendliness and ease of disposal due to its single-use design (Figure 2e). Its paper construction ensures lightweight comfort and easy application with medical-grade tape, maintaining secure placement on the skin [48].

PDMS has been utilized to create colorimetric epidermal patches, such as a chip modified with nonionic surfactants to improve hydrophilicity for efficient sweat collection. This PDMS chip is integrated with a low-cost paper-based sensor for glucose detection, supporting mass production and deployment in sports settings. Paired with smartphone optical color recognition software, it enables the real-time monitoring of low glucose levels in sweat during activities like long-distance running. However, the accuracy diminishes at higher glucose concentrations, limiting its usefulness for detecting elevated glucose levels [30].

## 4. Lactate Monitoring for Fatigue Assessment

Glucose breaks down to pyruvate through glycolysis. Aerobically, pyruvate becomes acetyl coenzyme-A via pyruvate dehydrogenase (PDH) for the Krebs cycle. Anaerobically, pyruvate turns into lactate via LDH, converting NADH + H^+^ to NAD^+^. Lactate fuels sustained exercise, with NAD^+^ aiding pyruvate regeneration in glycolysis [61,62]. Lactate accumulation can activate gluconeogenesis in liver and skeletal muscle cells, through which lactate is converted to glucose and released into the blood to drive additional glucose consumption during energy expenditure [63]. Monitoring lactate levels is crucial for assessing athletes’ physiological reactions, indicating aerobic and anaerobic capacities [64]. Steady-state lactate defines the anaerobic threshold’s peak intensity [65]. Lactate levels categorize exercise intensity into different zones. Zone 1, the low-intensity exercise zone, primarily utilizes fats as the main energy source rather than carbohydrates. Zone 2 involves moderate-intensity exercise, resulting in increased lactate production. Zone 3, the high-intensity exercise zone, is characterized by sustained elevated blood lactate levels throughout the activity [66,67].

Blood lactate ranges from 0.5 to 2.2 mM in healthy individuals [68]. Sweat is a primary fluid for noninvasive lactate monitoring, typically higher than blood [69]. Blood lactate can stabilize or decrease with constant exercise, while sweat lactate levels generally rise. The linear detection of sweat lactate spans 0–100 mM, with critical accuracy within 1–5 mM for exercise evaluation [69]. Blood and sweat lactate show a positive correlation [70,71,72] with blood changes detectable at exercise onset and sweat changes after ~1.60 s [73] due to the sweat collection time, rate, site, and method influencing correlation [74]. However, only working muscle area sweat shows lactate rising during exhaustive exercise [72].

To date, various fully integrated sweat lactate sensing systems suitable for in situ perspiration analysis have been developed (Table 2).

Electrochemical and colorimetric sensors were validated with elite athletes in controlled environments. A colorimetric biosensor’s efficacy was tested in a long-distance bicycling race, showing that the sensitivity, response times, and stability were unaffected by pH, temperature, or flow rate changes [70]. The correlations between sweat and blood lactate were discussed for continuous sports performance monitoring [70,74]. Most wearable glucose monitors are multifunctional, integrating lactate measurement using dual enzymes [24,35,36,37,40,43,45,47].

### 4.1. Electrochemical Devices

Electrochemical biosensing is widely used for lactate determination in sweat. Sensors measure lactate by detecting enzymatic oxidation–reduction reactions. The two main methods include the Lactate Dehydrogenase (LDH) method, which measures light absorption to gauge NADH formed during lactate conversion to pyruvate, and the Lactate Oxidase (LOx) method, which catalyzes lactate oxidation to pyruvate and hydrogen peroxide via FMN reduction.

Despite LDH’s accuracy, LOx is preferred in noninvasive monitors, as LDH requires the addition of exogenous NAD^+^ coenzyme [90].

#### 4.1.1. Patch-Type Devices

**Figure 3:**
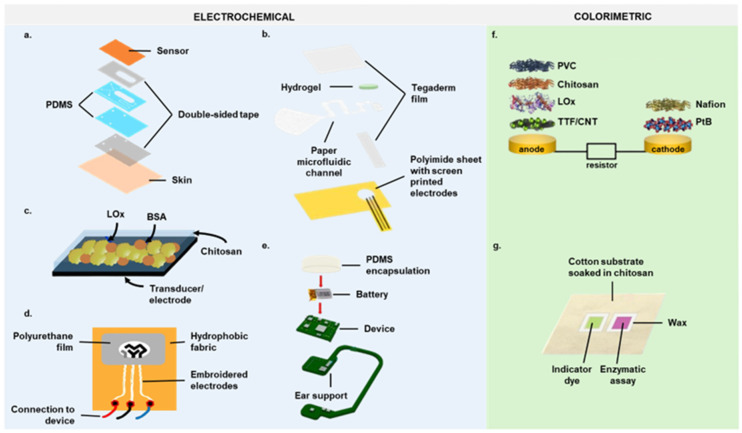
Structural anatomy of the most representative lactate biosensors. Diagram of (**a**) a patch-type sensor with microfluidics [73]; (**b**) a device consisting of an osmotic hydrogel, a paper microfluidic channel, a functionalized lactate sensor, and a flexible sheet with screen-printed electrodes [79]; (**c**) the working electrode coated by chitosan in a tattoo biosensor [80]; (**d**) a smart textile biosensor with embroidered electrodes on a hydrophobic fabric and three metal press buttons connected by wires to the electrochemical device [81]; (**e**) a smart wireless ear-worn device connected to an external battery and encapsulated in PDMS to protect the electronics [83]; (**f**) the layer makeup of the biofuel cell-based patch-type colorimetric sensor [47]; (**g**) a textile-based colorimetric sensor [89]. BSA, bovine serum albumin. PVC, polyvinyl chloride. PtB, platinum black. TTF, tetrathiafulvalene. CNT, carbon nanotube.

In epidermal patches, sweat sampling is improved by using low-dimensional sensing compartments and reducing channel hydrophilicity through silane functionalization. The fluidic channel captures and directs sweat through active sensing electrodes for real-time analysis. Ultra-small microfluidic devices with an amperometric lactate biosensor and other sensors for various biomarkers are co-fabricated on a flexible sensor array using screen-printed carbon masters and mounted on the skin [35,36,37,40,75,76]. To enhance the evaluation of the maximal lactate steady state during exercise, a poly(vinylidene fluoride)/Tetrapod-shaped ZnO/enzyme-modified nanocomposite film was developed. This biosensor leverages the piezoelectric surface coupling effect to monitor lactate concentration changes in real time under noninvasive conditions and operates without a battery [77]. Advanced microfluidic designs feature integrated circuits for signal processing paired with custom smartphone apps [70]. Additionally, a lactate sensor with a microchannel designed to trap air bubbles and prevent interference was developed to address measurement issues (Figure 3a) [73].

#### 4.1.2. Paper-Based Devices

Fully integrated sensors for continuous and selective sweat lactate measurement have been developed using flexible sensors and paper microfluidic platforms (Table 2). These systems transmit information wirelessly for real-time data analytics [43,78,79]. The design allows continuous sweat flow through flexible microneedle sensors in a microfluidic channel. The amperometric lactate sensor uses doped enzymes on a semipermeable copolymer membrane with outer polyurethane layers [78]. HIS paper’s low cost and convenience make it ideal for noninvasive electrochemical sensors for glucose and lactate detection. The three-dimensional diffusion path and hydrophilic properties of paper substrates enable efficient sweat collection and rapid diffusion [43]. To address insufficient sweating, Saha et al. developed a lactate monitoring platform that collects sweat over extended periods using hydrogels for osmotic extraction and paper microfluidic channels for sample evaporation (Figure 3b) [79].

#### 4.1.3. Tattoo-Based Devices

Epidermal patches lack elasticity, causing electrode detachment during physical exertion. To address this, flexible printed temporary transfer tattoo sensors were developed, which conform to the skin and maintain stability even during movement. Created using screen printing with dispersed carbon fibers, these tattoos can withstand extreme mechanical stresses [23,91,92]. The first noninvasive enzymatic tattoo electrochemical biosensor was designed to continuously monitor lactate levels in sweat [80]. It includes a mediated LOx working electrode and a biocompatible chitosan overlayer to prevent a reagent layer efflux onto the skin (Figure 3c). Tattoo biosensors were tested for adhesion under various strains like stretching, bending, and twisting. For in vivo evaluation, the biosensor was applied to the right deltoid of volunteers and interfaced with a hand-held electrochemical analyzer via a thin-film flexible connector secured with medical adhesives. Future developments will integrate the electronic interface, data processing, and wireless transmission of results [80].

#### 4.1.4. Fabric-Based Devices

Clothing-integrated sensors for tracking biochemical changes in sweat are a significant advancement in wearable technology. Traditional substrates like PET, PI, PDMS, and plastics, while effective, are non-degradable and can cause skin irritation with repeated use [93,94]. Textile-based substrates offer a sustainable alternative, providing lightweight, breathable, washable, and stretchable options for continuous wear [95]. An enzymatic textile-based biosensor was developed to monitor sweat lactate, using hydrophobic fabric to retain samples. Electrodes were printed on the fabric, with LOx mixed with a chitosan biopolymer immobilized over them (Figure 3d). This smart textile biosensor, integrated into chest belts and headbands, successfully monitored lactate in volunteers during exercise [81].

#### 4.1.5. Other Wearable Platforms

Biosensors are becoming compact, allowing integration into wearable accessories like eyeglasses, ear-worn devices, forehead bands, and smart bands [27,44,45,81,82,83,84,85,86,87,88]. For example, eyeglasses with a wireless lactate sensor on the nose bridge pads enable real-time monitoring [82]. Similarly, an ear-worn device measures lactate during exercise, sending data via Bluetooth to a mobile phone (Figure 3e) [83]. Since some devices may be uncomfortable for athletes, biosensors are often integrated into smart bands or sweat collectors [45]. Flexible substrates with wireless transmitters and capacitors create self-powered lactate analyzers that detect sweat lactate on various body locations (wrist, forehead, thigh, back) [84]. Additionally, iontophoresis-induced sweating aids in lactate sensing [27,85]. These wireless, easy-to-use devices are ideal for long-term monitoring, alerting users to health risks [44,86,87,88].

### 4.2. Colorimetric Devices

Electrochemical methods enable real-time monitoring data to be transmitted to external display devices, such as tablets or computers, allowing coaches to observe athletes’ physiological changes. However, a primary cause of monitoring errors in these sensors is that the sweating intensity and lactate concentration are independent [87]. Colorimetric approaches offer a simpler way for athletes to assess physiological changes in real time with minimal interruption to training, although their accuracy is inferior to electrochemical methods [74].

#### 4.2.1. Patch-Type Devices

New devices integrating chronometric microfluidic platforms with embedded colorimetric assays combine electronic and microfluidic functionality (Figure 3f). Systematic studies of electronics, microfluidics, and integration schemes highlight key design considerations and performance attributes [47]. These fully integrated, soft microfluidic systems feature networks of functionalized channels and reservoirs for sweat capture, routing, and storage, with spatially separated regions for analysis. Their soft mechanical properties, biocompatible materials, digitally analyzable colorimetric responses, and optimized structural, evaporative, and fluidic properties are crucial for effectiveness. These devices offer additional quantitative modes: digital image capture analysis for simple quantitation and direct electronic readout [24].

#### 4.2.2. Fabric-Based Devices

A fiber-based colorimetric sensor was developed to detect pH and lactate in sweat simultaneously (Figure 3g) [89].

This sensor consists of three layers on cotton fabric: chitosan as the first layer, a mix of bromocresol green and methyl orange as pH indicators for the second layer, and sodium carboxymethyl cellulose as the third layer. A hydrophobic material was screen-printed to separate the pH and lactate detection zones, with LOx molecules used for lactate detection [89]. Tested on three volunteers during a 30 min jog, the sensor effectively differentiated their fitness levels and potential muscle fatigue. Its textile nature allows integration into items like tights, wristbands, headbands, and wearable devices for nonintrusive real-time athletic performance monitoring [89].

## 5. Electrolytes and pH Monitoring for Hydration and Muscle Fatigue Assessment

In addition to metabolites like glucose and lactate, ion concentration profiles in sweat reveal personal dynamic patterns during sports activities [96]. Sodium (Na^+^) and chloride (Cl^−^) are the most abundant electrolytes in sweat. Their excretion is influenced by exercise intensity and indicates electrolyte imbalance [97]. It serves as a biomarker for monitoring athletic performance, especially in hot and humid environments, where sweat losses can impair physical and mental well-being [23]. An increased dietary Na^+^ and Cl^−^ intake is necessary to compensate for losses after intense exercise to maintain electrolyte balance for accelerating recovery and minimizing soft tissue injuries from dehydration [98,99].

Other key electrolytes in sweat include potassium (K^+^) and ammonium (NH_4_^+^) ions. The K^+^ efflux rate is directly proportional to the exercise intensity, providing insights into training intensity [100]. Monitoring K^+^ levels in sweat can thus inform training intensity, although the mechanism linking the K^+^ efflux rate and sweat rate remains unclear [8]. Low K^+^ sweat levels can indicate dehydration, which can impair the electrical impulse communication between cells in addition to inducing muscle cramps [101].

NH_4_^+^ is a small molecule whose concentration increases during exercise when the body transitions from an aerobic to an anaerobic state. Studies have demonstrated a direct correlation between NH_4_^+^ levels in sweat and its concentration in plasma, where plasma ammonia (NH_3_) is the primary source of NH_4_^+^ in sweat [102]. NH_4_^+^ levels reflect protein breakdown, providing critical insights into the transition from aerobic to anaerobic metabolism when carbohydrate sources are depleted during high-intensity exercise [103]. Hence, monitoring NH_4_^+^ levels could therefore offer valuable insights for optimizing sports performance.

Additionally, calcium (Ca^2+^) is crucial for human metabolism and mineral balance [104]. Significant fluctuations in Ca^2+^ levels in biofluids can harm organs and systems. However, wearable sensors for monitoring Ca^2+^ levels in body fluids are underexplored.

Sweat pH is vital for exercise monitoring and is linked to electrolyte balance. Sweat typically ranges from pH 4.5 to 7.0 [105], with higher pH indicating increased sweat rates. Elevated pH correlates with higher Na^+^ levels, signaling dehydration severity and exercise intensity [106,107]. During exercise, sweat pH rises primarily due to two reasons. Firstly, prolonged activity leads to significant accumulation of bicarbonate (HCO_3_^−^) on the skin, elevating sweat pH [108]. Secondly, NH_3_ in sweat decreases as it converts to NH_4_^+^, which accumulates due to its reduced diffusion across cellular membranes compared to NH_3_, further increasing sweat pH [105]. Therefore, monitoring sweat pH can potentially correlate with acid build-up in muscle cells during exercise, contributing to muscle fatigue.

Wearable and autonomous devices capable of alerting users to electrolyte loss and the need for replenishment during prolonged indoor and outdoor physical activities have been designed [44,109] (Table 3). On-body measurements from these wearable ion sensors have been validated using ion chromatography, atomic absorption, and inductively coupled plasma mass spectrometry methods [76,96,110,111,112].

### 5.1. Electrochemical Devices

Three main technologies for wearable sweat ion sensors include titration devices, conductivity measurements, and potentiometric sensors. Wearable titration sensors require subsequent analysis on a separate instrument [124], whereas conductivity sensors and potentiometric devices are easily miniaturized [125]. Potentiometric devices, based on a well-established technique, offer a straightforward approach to sweat ion sensing. They measure ion concentration by analyzing the electrochemical potential difference between a working electrode and a reference electrode, which varies logarithmically with ion concentration. Signal conditioning circuitry aids in measurement accuracy [126]. Specifically, ion-selective electrodes (ISEs) form the basis of this method. Traditional ISEs include a membrane-based ion-selective electrode and a reference electrode, both requiring an internal solution for stability and sensitivity, complicating fabrication, and limiting miniaturization. Solid-contact ISEs, where an ion-selective membrane is applied directly onto a solid metal wire, were developed to address these challenges [127]. The initial issues with coated-wire sensors’ reproducibility were resolved by using conducting polymers as ion-to-electron transducers [92,128,129]. Traditional ISEs are typically made from rigid materials like glass, hindering integration on curved surfaces. For wearable applications, sensor arrays must interface with FPCBs for signal processing, conditioning, and wireless transmission [44,130,131]. Selectivity is crucial, necessitating an evaluation of how major interfering electrolytes impact sensor performance [112]. Potentiometric sensors offer simplicity for on-body electrolyte and pH measurement; however, the equilibration between reference and test solutions poses a significant error source, limiting measurement accuracy over time [119].

#### 5.1.1. Patch-Type Devices

**Figure 4:**
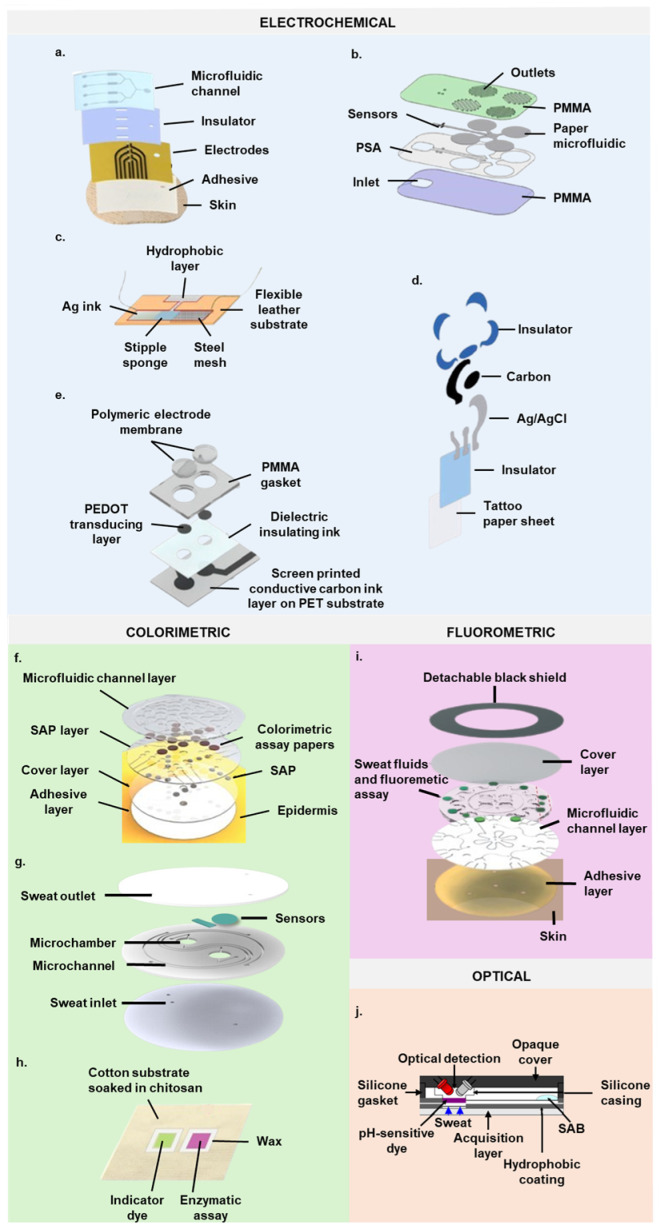
Structural anatomy of the most representative electrolytes and pH biosensors. Schematic illustration of (**a**) the different layers of a microfluidic epidermal patch [76], (**b**) a paper microfluidic chip [78], (**c**) a potentiometric ion sensor embedded into a flexible leather substrate [115], (**d**) the different layers of a potentiometric tattoo sensor [91], (**e**) the individual components of the PEDOT ion-selective electrode [117], (**f**) a colorimetric epidermal microfluidic device [121], (**g**) a colorimetric paper-based microfluidic chip [30], (**h**) a textile-based colorimetric sensor [89], (**i**) a skin-interfaced fluorometric microfluidic device [111], (**j**) a pH sensor with an optical detection system [123]. PMMA, Poly (methyl methacrylate). PSA, pressure-sensitive adhesive. SAB, surface-activated bonding. SAP, superabsorbent polymer.

Continuous sweat ion collection, transport, and analysis are achieved with pump-free epidermal microfluidic devices [96,97,113]. A salt bridge integrated into a thin film Cl^−^ sensor minimizes equilibration-induced measurement errors, ensuring stable measurements over extended periods with minimal concentration drift despite small sample volumes [97]. Biocompatible threads facilitate sample collection via capillary absorption, delivering perspiration into a hydrophilic microfluidic channel with Na^+^ electrodes to maintain continuous flow [113]. pH, Na^+^, Cl^−^, and K^+^ sensors integrated into stretchable materials or a flexible plastic substrate prevent sweat contamination and evaporation, with sweat flow controlled by pressure induced by secreted sweat in a flexible sampling cell [96,114]. These patches autonomously analyze sweat, interfacing with an FPCB for on-site signal conditioning, analysis, and wireless transmission [114]. Additionally, microfluidic patches enable simultaneous amperometric glucose or lactate and potentiometric ion sensing (Figure 4a) [39,76]. Floating potentiometric circuits in these patches eliminate signal interference from adjacent amperometric transducers, ensuring crosstalk-free signal collection and wireless transmission [76]. Wang et al. developed a microchip for measuring glucose, lactate, Na^+^, and K^+^ with sensors resistant to deformation and flexible materials, ensuring reliable wearability on the forehead during physical activities with minimal detachment errors. Data from the chip are transmitted to a separate application for analysis [36].

#### 5.1.2. Paper-Based Devices

Paper’s natural wicking properties enable effective sweat sampling, mitigating issues with sweat accumulation that can lead to inaccurate measurements over extended periods [44]. A microfluidic paper-based device was developed to simultaneously measure the pH, Na^+^ ion concentration, and lactate levels. This device incorporated a flexible sensor array within paper microfluidic channels. Sweat was drawn into the paper channel through capillary action via small windows, ensuring continuous absorption and preventing accumulation. Exit ports facilitated sweat evaporation, maintaining a constant flow across the sensor surface. The device featured potentiometric pH and Na^+^ ion sensors, along with amperometric lactate sensors. The pH sensor utilized a highly sensitive iridium oxide membrane, the Na^+^ ion sensor employed a Poly(3,4-ethylenedioxythiophene) (PEDOT) polymer membrane, and the lactate sensor included enzymes on a semipermeable copolymer membrane (Figure 4b) [78]. This wearable electronic sensor was completed with wireless readout electronics. This wearable electronic sensor was integrated with wireless readout electronics for real-time monitoring.

#### 5.1.3. Tattoo-Based Devices

Potentiometric ISEs were developed on temporary transfer tattoo paper for direct epidermal pH and electrolyte measurements. These tattoo-based devices were created by integrating solid-contact ISEs with commercially available temporary transfer tattoo paper using screen printing techniques (Figure 4d) [91,92,109]. These skin-worn, noninvasive tattoo-like sensing devices were realized by using ion-selective polymeric membranes and a solid-state reference electrode, combining the advantages of tattooed electrochemical sensors with the unique features of solid-state potentiometric sensors, such as extremely low power consumption, simplicity of operation, and wide linear dynamic ranges [91,109,132]. The resulting tattoo-based potentiometric sensors showed minimal carryover effects and high resilience to various mechanical deformations of the human epidermis [91,92,109]. When paired with a custom-designed Bluetooth-enabled wireless wearable transceiver housed in an adjustable armband, these sensors enabled continuous, noninvasive monitoring of sweat ion levels directly on the human skin [109].

#### 5.1.4. Fabric-Based Devices

Recently, a novel method for real-time quantitative analysis of Na^+^ in human sweat has garnered considerable attention. This method utilizes printed electrochemical sensors on textiles, optimizing both sweat collection and analysis within a single, integrated wearable platform (Figure 4c) [110,115]. A miniaturized potentiometric cell was fully integrated into wearable items like belts or headbands. Sweat is continuously drawn from the skin to a sensing surface and then to a storage area using a fabric pump [110,115]. The sensing material employed was sodium manganese oxide, selected for its capacity to incorporate Na^+^, electrical conductivity, stability, and cost-effectiveness [115].

#### 5.1.5. Other Wearable Platforms

Smart bands for real-time sweat ion monitoring integrate vertically arranged components: sampling system, electrodes, circuitry, and battery. Solid-contact ISEs were developed with either PEDOT or poly(3-octylthiophene-2,5-diyl) (POT) as the conductive polymer (Figure 4e) [117]. Sweat enters via a sampling orifice, passes over ion-selective and reference electrodes, and moves into a storage area with high-capacity adsorbent material. Capillary action drives liquid movement, adjustable via channel width, ensuring sensitivity and selectivity to sweat interferents [116,117]. Potentiometric signals are processed by integrated electronics, digitized, and transmitted via Bluetooth to a remote base station [116,117,118,119]. A mechanically flexible smart band with an integrated sensor array simultaneously analyzes glucose, lactate, Na^+^, and K^+^ in sweat, requiring no external processing. Plastic-based sensors interface with the skin, while silicon integrated FPCBs handle signal processing [44]. These platforms support a variety of personalized physiological monitoring applications [44,85]. Additionally, Sempionatto et al. developed sensor eyeglasses with an amperometric lactate sensor and a potentiometric K^+^ sensor on the nose pads, while Gil et al. created an ear-worn device with a pH sensor on gold-plated electrodes [82,83]. Tests confirmed accurate simultaneous recordings of lactate, K^+^, and pH without interference [82,83].

Real-time Ca^2+^ detection in sweat is underexplored. However, an electrochemical device for continuous Ca^2+^ and pH monitoring in sweat, using a disposable flexible sensor array with a printed circuit board, was designed for arm or forehead use. This system showed an inverse relationship between Ca^2+^ concentration and sweat pH [112].

### 5.2. Colorimetric Devices

Colorimetric biosensors offer significant advancements in noninvasive sweat electrolyte and pH analysis, providing better control over sweat sampling interfaces. These devices use flexible bands with superhydrophobic–superhydrophilic microarrays in a thin, closed microfluidic system to directly collect sweat from skin pores. They incorporate biocompatible adhesives and are stretchable, allowing comfortable placement on various body parts without causing irritation [24,46,120,121,122]. Similar to electrochemical patches, many colorimetric patches can simultaneously monitor metabolites like glucose and lactate, along with ions and pH (Figure 4g,h) [24,30,46,89,120].

#### Patch-Type Devices

Patch-type devices eliminate the need for direct skin electrode contacts, reducing irritation and noise from motion artifacts. These ultrathin, breathable, and stretchable sensors closely match the skin’s properties, ensuring minimal disruption and enabling long-term health monitoring. The substrates fill with body fluids, causing colorimetric changes [120]. Developed by doping silicon substrates with colorimetric indicators, these devices are sensitive to pH values and have been tested with standard buffer solutions [120]. They use inductive coupling schemes, potentially compatible with near-field communication (NFC) systems in portable electronic devices [120,122]. A real-time microfluidic device for monitoring sweat Cl^−^ used water-actuated valves with super absorbent polymers to isolate sweat reservoirs, preventing backflow and allowing air ventilation. This design featured hydrophobic and hydrophilic channel surfaces in a multilayer setup for stable and precise sensing. The device provided colorimetric readouts of time-sequenced sweat samples, utilizing assay chemistry with noninteracting ions and surfactants to stabilize color development for accurate Cl^−^ analysis across various concentrations (Figure 4f) [121]. Its conformal properties enabled watertight seals to the skin discreetly. A waterproof platform adhered softly to the skin for real-time monitoring of local sweat Cl^−^ concentration in aquatic athletes via underwater sweat collection. Tested in salt water, it showed robust adhesion, proper filling, and reliable operation in extreme conditions [122]. These devices routed collected sweat through separate channels and reservoirs, enabling multiparametric sensing of glucose, lactate, pH, and electrolytes [24,46].

### 5.3. Fluorometric Devices

A fluorescence-based method for analyzing sweat electrolytes offers high sensitivity using specific probes that react with ions and emit fluorescence when excited by light. Sekine et al. developed a wearable biosensor for in situ measurement of sweat Cl^−^ and Na^+^ levels. This device includes microchannels and valves directing sweat to microreservoirs with fluorometric probes (Figure 4i). As sweat flows through, the probes react with ions, altering fluorescence intensity. Detection and analysis involve photographing the biosensor with a smartphone equipped with digital imaging software. The results were validated against traditional lab methods, showing effective ion concentration determination [111]. However, these platforms require interruptions for data collection, limiting continuous real-time monitoring during athletic activities.

### 5.4. Optical Devices

A textile-integrated optical sensor for sweat pH monitoring was developed within a waistband platform [123]. This device houses the pH sensor, electronics, and a reference patch in absorbent fabric (Figure 4j). Sweat is directed through a predefined path into the platform, where the sensor is enclosed, with only the collection layer contacting the skin. The pH-sensitive reagent on the textile changes color (yellow to blue) within the pH range of 4–7. Quantitative pH measurements utilize a paired emitter–detector dual LED setup positioned over the fabric channel within the waistband’s rubber gasket. The system demonstrated real-time monitoring capabilities during exercise through in vitro and on-body trials, wirelessly transmitting data to a laptop for analysis [123].

## 6. Cortisol Measures to Assess Stress

Cortisol, a hormone secreted by adrenal glands in response to stress, affects metabolism, electrolytes, and blood pressure [133]. When present in excess, it functions as a catabolic hormone, increasing the availability of fuel substrates by mobilizing glucose, free fatty acids, and amino acids from endogenous stores. These actions are physiologically important during periods of stress, such as fasting and exercise [134]. In particular, the acute cortisol response to exercise is highest when the overall stress of the training (in terms of volume and/or intensity) is elevated [135]. Wearable sensors for cortisol offer the potential for stress monitoring in athletes, supporting well-being. Sweat cortisol levels (8–50 ng/mL) correlate strongly with serum levels [136,137]. Current sensors have been tested in buffer or artificial sweat, with limited studies on real sweat dynamics [138]. Four patches for real-time cortisol monitoring during physical activity have been developed [136,138,139,140] (Table 4).

### 6.1. MIP-Based Patch-Type Devices

**Figure 5:**
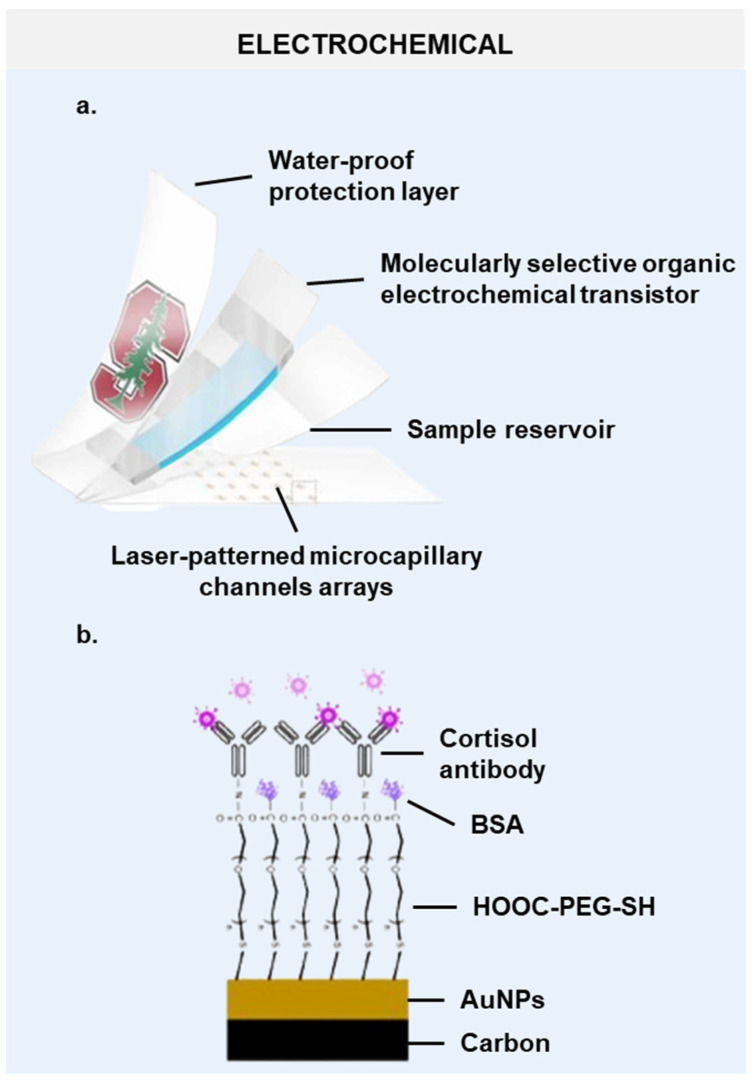
Structural anatomy of the most representative cortisol biosensors. Schematic illustrations of (**a**) a patch-type cortisol sensor composed of multiple layers [138] and (**b**) a flexible electrochemical patch with cortisol antibodies immobilized on its surface [139]. AuNPs, Au nanoparticles. BSA, bovine serum albumin. HOOC-PEG-SH, thiol-polyethylene glycol-carboxyl.

Organic electrochemical transistors (OECTs) excel at converting biological ion signals to electrical ones due to their high gain at low voltages [141,142]. However, they are not naturally suited for cortisol detection, as cortisol is uncharged at physiological pH. Molecularly imprinted polymers (MIPs) offer a solution by acting as versatile, selective receptors [143]. MIPs mimic natural receptors, showing high affinity and specificity for target molecules, ideal for sensors and separation processes [140,144]. MIPs are synthesized through the copolymerization of monomers, crosslinkers, and the template molecule. The removal of the template leaves behind cavities within the polymer network that match the shape and size of the template. These membranes promise effective, cost-efficient, stable performance in sweat-based wearable sensors across various conditions [138]. Flexible MIP-based electrochemical sensors have been developed specifically for cortisol detection in human sweat, with an MIP-based membrane guiding cortisol to the OECT sensing channel for precise detection (Figure 5a) [138,140].

### 6.2. Patch-Type Immunosensors

A rapid on-site method for detecting sweat cortisol involves electrochemical immune-sensing techniques using a wireless, flexible, epidermal detection system [136,145,146,147]. These devices utilize NFC technology for wireless energy supply and data transmission. This design allows for a fully flexible structure, incorporating only a small NFC chip and antenna, eliminating rigid batteries or wires. The electrochemical immunosensor includes screen-printed electrodes with immobilized cortisol antibodies, integrated into a patch along with the detection circuit on a flexible substrate (Figure 5b). This setup ensures comfortable attachment to the skin. These cortisol sensors exhibit excellent sensitivity, linearity, and selectivity for continuous the in situ detection of sweat cortisol, effectively capturing circadian cortisol rhythms under various conditions, demonstrating their capability for accurate and continuous monitoring [136,139].

## 7. Multimodal Devices

To date, many wearable devices for monitoring sweat composition can analyze multiple biomarkers simultaneously, providing a comprehensive profile (Table 5). Given sweat’s complexity, the simultaneous screening of biomarkers requires integrated systems for accurate measurements [44].

Most multifunctional biosensors can measure two biochemical parameters, often alongside physiological ones. For instance, devices measure glucose and lactate using various technologies such as paper-based platforms, forehead bands, and carbon-based microchips [35,37,40,43,45]. Hybrid devices integrate electronic and microfluidic functions with colorimetric assays to monitor these metabolites [47]. Textile-based colorimetric sensors and wireless electrochemical devices in forms like ear-worn devices or forehead/thigh bands can simultaneously detect sweat pH and lactate [83,85,89]. Instead, an eyeglasses-based platform integrates wireless multiplexed chemical sensing for the real-time monitoring of sweat lactate and K^+^ ions [82]. The other pairs of biomarkers monitored include pH and electrolytes, where colorimetric systems offer flexibility and stretchability, while potentiometric ion sensors are preferred for reproducibility and accuracy [96,112,120]. Two devices combine glucose monitoring with pH measurements using electrochemical or colorimetric methods [30,39]. In one case, an electrochemical biosensor for glucose modified its working electrode with a Prussian blue mediator and a glucose oxidase enzymatic layer, alongside a pH sensor using the same conductive pattern with pH-selective and reference membranes for potentiometric readings [39]. In another instance, rapid detection test strips enabled simple and low-cost colorimetric sensing of glucose and pH using commercial strips for hydrogen peroxide and pH determination [30].

Other devices are designed to measure three biomarkers simultaneously in sweat samples. Fully printed wearable microfluidic devices analyze lactate, electrolytes, and pH, or lactate, electrolytes, and glucose [36,44,76,78]. Superwettable colorimetric sensing bands detect sweat glucose, electrolytes, and pH in situ, utilizing superhydrophilic microwells for continuous sweat collection and smartphone-based image recognition for colorimetric detection [46]. Finally, a microfluidic patch with four quantitative colorimetric assay reagents assesses pH, glucose, lactate, and Cl^−^ concentrations through enzymatic or chromogenic reactions [24].

In this context, eudaimonic technology represents an exciting frontier in the realm of human well-being. Eudaimonic interaction design is an approach in human–computer interaction that aims to create digital experiences promoting long-term well-being and personal fulfillment rather than just immediate pleasure or efficiency. Grounded in positive psychology, it draws on the concept of eudaimonia—a Greek term referring to the flourishing life and realization of one’s true potential. Scientifically, this involves designing interfaces and interactions that encourage users to engage in meaningful activities, fostering personal growth, autonomy, competence, and social connectedness. Unlike traditional hedonistic design, which focuses on maximizing short-term satisfaction, eudaimonic design targets deeper, intrinsic needs that contribute to sustained well-being and a sense of purpose [148].

When applied to wearable technology for athletes, eudaimonic technology can extend beyond optimizing physical performance to also support athletes’ holistic well-being, encompassing their mental, emotional, and psychological health.

While wearable devices such as biosensors already track physiological and biochemical parameters like glucose, lactate, electrolytes, pH, and cortisol to enhance training and recovery, eudaimonic technology would shift the focus toward fostering overall mental and emotional resilience, helping athletes thrive both on and off the field [148,149].

## 8. Future Research Directions

From a human-centered design perspective, technological evolution presents exciting opportunities for exploration. Key questions arise, such as how technology can achieve a balance between autonomy and maintaining a high level of human satisfaction.

This challenge, while complex, invites exploration into how emerging technologies can empower individuals without compromising well-being. A potential direction could involve designing systems that offer users greater control over their interactions with technology, ensuring that autonomy is maintained while also prioritizing user satisfaction through intuitive interfaces and personalized feedback. Additionally, fostering transparency in how technology collects and uses data could contribute to a sense of trust and fulfillment. As technology becomes increasingly autonomous, integrating mechanisms for ongoing human oversight might be crucial to safeguard satisfaction. One approach could be to integrate user feedback loops that adapt the technology to individual preferences and well-being, ensuring that both autonomy and satisfaction remain aligned.

Furthermore, it prompts reflection on the qualities that define meaningful interactions with technology. Eudaimonic technology, in this context, represents an exciting frontier—focusing on creating experiences that prioritize the quality of interaction and the overall process, rather than solely on outcomes [148,149].

A further avenue for exploration is how AI can be utilized to achieve this balance in future technologies, particularly by designing systems that empower users to make informed decisions while maintaining control over their data and privacy.

The latest improvement in artificial intelligence (AI) can be integrated into athlete management and can further enhance the utility of these wearables by correlating data on metabolic rates, dietary intake, and fitness exercises [150]. Sweat sensor data, alongside nutritional information and exercise routines, provide a comprehensive overview of an athlete’s physiological state. This correlation allows for more personalized and effective training programs, tailored dietary recommendations, and optimized recovery strategies. By understanding how an athlete’s metabolism interacts with their diet and physical activities, AI-driven systems can help in fine-tuning performance and maintaining peak conditions.

In addition to these post-measurement applications, AI holds substantial potential for enhancing the biosensing process itself. Currently, most devices, as shown in Table 5, measure only two or three parameters simultaneously. By leveraging AI-driven data extraction, it may be possible to increase the number and accuracy of biomarkers that biosensors can detect concurrently. Advanced AI algorithms can improve the signal-to-noise ratio, reducing interference and enabling more parameters to be monitored with existing hardware. Furthermore, AI can enhance sensor calibration, adjusting in real time to factors like sweat rate, temperature, and individual physiology, thereby creating a more comprehensive and accurate picture of the user’s state. These developments could lead to a new generation of “smart” biosensors capable of tracking multiple biomarkers simultaneously, without additional hardware, thus enhancing both efficiency and user experience.

Researchers like Jörs and De Luca [151] have proposed design principles for Eudaimonic User Experiences (EUXs) that could be integrated into sweat sensor wearables. These principles emphasize fostering behaviors such as learning, intrinsic motivation, and cognitive engagement, which contribute to psychological well-being. AI can assist in implementing these design rules by providing informative feedback, enhancing data privacy, and avoiding manipulative design patterns. This approach supports user autonomy and environmental mastery, laying the groundwork for interactive systems that contribute to eudaimonic well-being.

Moreover, AI-driven sweat sensors could be designed to prioritize features that encourage users to reflect on their health and set meaningful goals. Unlike traditional wearables that focus on metrics like steps or calories, AI-enhanced devices could help users understand the impact of their daily habits on their overall life goals and well-being. By promoting mindfulness, self-reflection, and positive relationships, these devices would align with eudaimonic principles and foster a deeper connection between users and their life aspirations. Ultimately, the integration of AI into sweat sensor eudaimonic design, combined with the correlation of metabolic, diet, and fitness data, holds the potential to create.

Future directions could include exploring how AI systems can be continually refined to better adapt to users’ evolving needs, ensuring that both autonomy and satisfaction are not only preserved but enhanced over time.

## 9. Conclusions

Athlete activity and biometric data analysis via wearable devices now include monitoring interactions and well-being, aiming to improve performance and enable systematic coaching [152]. While most devices track physiological data, only a few measure biochemical markers, which are essential for tailored training and recovery, particularly through sweat, which contains electrolytes, metabolites, and hormones [54]. Devices must perform reliably in diverse conditions, including aquatic environments [153].

Monitoring sweat glucose and lactate during exercise optimizes energy and endurance [154], as glucose tracking is vital for identifying glycogen depletion in endurance sports [121,155,156]. Lactate levels indicate exercise intensity, showing when fatigue sets in or when recovery is underway [62,65].

Tracking sweat electrolytes and pH reveals hydration levels and muscle function, preventing dehydration or overhydration [109]. Electrolyte imbalances can cause cramps and fatigue [98,101], and pH imbalances affect metabolism and enzyme function, leading to fatigue [97,105]. Monitoring cortisol levels can also indicate stress, with prolonged elevation potentially causing chronic fatigue [133,138].

Sweat analysis uses electrochemical methods to convert analyte data into electrical signals, ideal for multi-channel detection [44,91,112]. These devices, though, face signal interference during exercise. Alternatively, colorimetric devices use assay reagents to measure metabolites, making them effective in sports despite limitations in available reagents [15,24,118,122,157,158,159].

**Figure 6:**
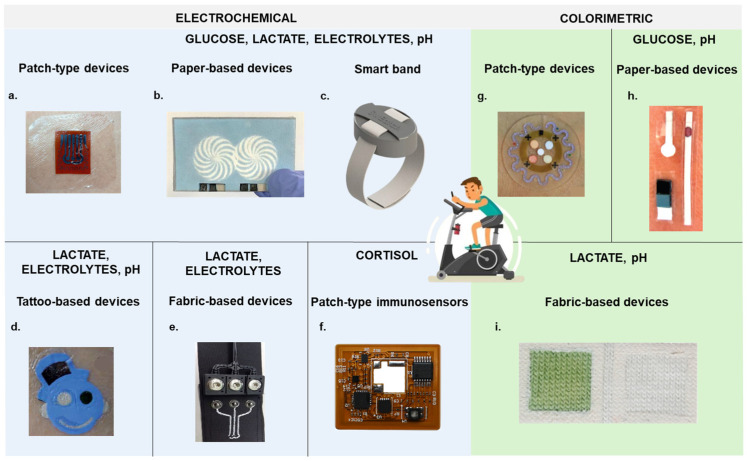
Overview of the main types of wearable devices for monitoring biochemical markers. The most representative devices are the electrochemical ones (**a**–**f**). (**a**) Photograph of an epidermal patch worn on a subject’s chest [37]. (**b**) Optical image of a HIS paper-based Ti_3_C_2_T_x_/MB electrode under original state [43]. (**c**) Photograph of a smart band [117]. (**d**) Image of an epidermal ISE tattoo applied to cubital fossa [92]. (**e**) Illustration of embroidered textile electrodes integrated into a belt [81]. (**f**) Photograph of a wireless, battery-free, flexible patch for in situ cortisol detection [139]. Less diffused are the colorimetric devices (**g**–**i**). (**g**) Photograph of an epidermal microfluidic biosensor integrated with flexible electronics mounted on the forearm [24]. (**h**) Photograph of a device made of paper strips mounted on the forehead [48]. (**i**) Optical image of a cotton textile-based colorimetric sensor directly patched on the abdomen of volunteers [89].

Current sweat analysis devices include epidermal patches, temporary tattoos, and fabric-based sensors that adhere to the skin, contrasting with rigid wearables that can yield lower-quality data (Figure 6) [35,89,91,122]. Biosensors have shown practical utility in moderate exercise, with results comparable to lab analyses [54].

Integrating eudaimonic design into wearable devices for sweat monitoring can enhance the user experience by focusing on long-term well-being. These devices could track real-time biochemical data while offering insights into how training impacts fitness and overall health. The design could include features that set meaningful goals based on an athlete’s personal growth, encourage autonomy by allowing them to reflect on their progress over time, and facilitate social connections by sharing achievements with coaches or training communities.

The ultimate goal is to develop a multimodal, noninvasive device that measures parameters like heart rate, glucose, lactate, pH, and cortisol, supporting the continuous tracking of fatigue, hydration, and stress. The device will feature stretchable materials and transmit data in real time for AI processing [69].

Wearables designed to monitor training effects can inform tailored programs for individual athletes and broader strategies within specialties [160]. Tracking each athlete’s load response allows for safe, progressive training plans, refined through real-time data and biomarker-based improvements [161]. This is particularly beneficial for athletes with metabolic conditions, such as diabetes [162]. AI-powered devices designed with eudaimonic interaction could enhance personalized exercise programs, optimizing exercise type, duration, and intensity.

## Figures and Tables

**Table 1:** Noninvasive sweat glucose monitoring devices in physical activity performance studies.

Biosensor Method	Wearable Platform	Exercise Modalities Tested	Human Trials	Refs.
Electrochemical	Patch-type devices	Stationary cycling	-	[35,36,37,38,39,40,41]
	Paper-based devices	Stationary cycling	-	[42,43]
	Forehead band Smart band	Stationary cycling		[44,45]
		Skipping rope	Skipping rope for a clinical study monitoring four healthy subjects over a period of one month	
		Burpee test		
		Outdoor running		
Colorimetric	Patch-type devices	Stationary cycling	12 cyclists competed in an outdoor race	[24,46,47]
		Running		
	Paper-based devices	Stationary cycling Running	-	[30,48]

**Table 2:** Noninvasive sweat lactate monitoring devices in physical activity performance studies.

Biosensor Method	Wearable Platform	Exercise Modalities Tested	Human Trials	Refs.
Electrochemical	Patch-type devices	Stationary cycling Running Kayaking	-	[35,36,37,40,70,73,75,76,77]
	Paper-based devices	Stationary cycling Running	-	[43,78,79]
	Tattoo-based devices	Stationary cycling	-	[80]
	Fabric-based devices	Running	-	[81]
	Smart band Forehead band Chest belt Ear-worn device Eyeglasses	Stationary cycling Running Squat	-	[27,44,45,82,83,84,85,86,87,88]
Colorimetric	Patch-type devices	Stationary cycling	12 cyclists competed in an outdoor race	[24,47]
		Jogging		
	Fabric-based devices	Jogging	-	[89]

**Table 3:** Noninvasive sweat electrolytes and pH monitoring devices in physical activity performance studies.

Biomarker	Biosensor Method	Wearable Platform	Exercise Modalities Tested	Human Trials	Refs.
Electrolytes	Electrochemical	Patch-type devices	Stationary cycling Running	-	[36,76,96,97,113,114]
		Paper-based devices	Stationary cycling Running		[78]
		Tattoo-based devices	Stationary cycling	-	[91,109]
		Fabric-based devices	Stationary cycling Running		[110,115]
		Forehead band Smart band Sensor belt Eyeglasses	Stationary cycling Indoor running Outdoor running	-	[44,82,112,116,117,118,119]
	Colorimetric	Patch-type devices	Stationary cycling	12 cyclists competed in an outdoor race	[24,46,120,121,122]
			Swimming		
	Fluorometric	Patch-type devices	Running on an elliptical trainer	-	[111]
pH	Electrochemical	Patch-type devices	Stationary cycling	-	[39,76,96]
		Paper-based devices	Stationary cycling Running	-	[78]
		Tattoo-based devices	Stationary cycling	-	[92]
		Ear-worn device Forehead band	Stationary cycling	-	[83,85,111]
	Colorimetric	Patch-type devices	Stationary cycling	12 cyclists competed in an outdoor race	[24,46,120]
		Paper-based devices	Running	-	[30]
		Fabric-based devices	Jogging	-	[89]
	Optical	Waistband	Stationary cycling	-	[123]

**Table 4:** Noninvasive sweat cortisol monitoring devices in physical activity performance studies.

Biosensor Method	Wearable Platform	Exercise Modalities Tested	Human Trials	Refs.
Electrochemical	MIP-based patch-type devices	Outdoor running	-	[138,140]
	Patch-type immunosensors	Stationary cycling	-	[136,139]

**Table 5:** Multimodal devices for noninvasive monitoring of sweat biomarkers in physical activity performance studies.

Glucose	Lactate	pH	Electrolytes	Cortisol	Refs.
					[35,37,40,43,45,47]
					[83,85,89]
					[96,112,120]
					[30,39]
					[82]
					[76,78]
					[36,44]
					[46]
					[24]

## Data Availability

Not applicable.
